# Identification of Candidate Allosteric Modulators of the M1 Muscarinic Acetylcholine Receptor Which May Improve Vagus Nerve Stimulation in Chronic Tinnitus

**DOI:** 10.3389/fnins.2017.00636

**Published:** 2017-11-14

**Authors:** Tijana Bojić, Vladimir R. Perović, Milan Senćanski, Sanja Glišić

**Affiliations:** ^1^Laboratory of Radiobiology and Molecular Genetics, Institute of Nuclear Sciences Vinča, University of Belgrade, Belgrade, Serbia; ^2^Center for Multidisciplinary Research, Institute of Nuclear Sciences Vinča, University of Belgrade, Belgrade, Serbia

**Keywords:** tinnitus, muscarinic allosteric agonists, M1 receptor, vagus nerve stimulation, *in silico* analysis, information spectrum method

## Abstract

Chronic tinnitus is characterized by neuroplastic changes of the auditory cortex. A promising method for therapy of chronic tinnitus is vagus nerve stimulation (VNS) combined with auditory stimulation. The principle of VNS is reversal of pathological neuroplastic changes of the auditory cortex toward physiological neural activity and synchronicity. The VNS mechanism of action in chronic tinnitus patients is prevailingly through the muscarinic neuromodulation of the auditory cortex by the activation of nc. basalis Meynerti. The aim of this study is to propose potential pharmaceutics which may improve the neuromodulatory effects of VNS. The working hypothesis is that M1 receptors have a dominant role in the neural plasticity of the auditory cortex. We propose that allosteric agonists of the muscarinic receptor type 1 (M1) receptor could improve specificity and selectivity of the neuromodulatory effect of VNS on the auditory cortex of chronic tinnitus patients even in the circumstances of lower acetylcholine brain concentration. This intervention would also reinforce the re-learning process of tinnitus (sub)networks by acting on cholinergic memory and learning mechanisms. We performed *in silico* screening of drug space using the EIIP/AQVN filter and selected 50 drugs as candidates for allosteric modulators of muscarinic receptors. Further filtering of these compounds by means of 3D QSAR and docking revealed 3 approved drugs—bromazepam, estazolam and flumazenil as the most promising candidates for combined chronic tinnitus therapy. These drugs should be further evaluated by biological tests and clinical trials.

## Introduction

Tinnitus, the perception of phantom sound, is a debilitating condition with notable prevalence: approximately 10–15% of the general population experience tinnitus and for 7 million this is a debilitating condition (Geven et al., 2014). The Royal National Institute for Deaf People estimates that 13 million people in Western Europe and USA seek medical assistance for their tinnitus symptoms (Vio and Holme, 2005). Demographic development and increase of occupational and ambient noise support the view that this problem will be even more prevalent in the future. In spite of the fact that more than 4 million prescriptions are written for the treatment of tinnitus, there is not a single drug approved by the FDA specific for the treatment of tinnitus. The estimates are that a potential tinnitus drug could have a product value of more than $600 million in the first year after its release (https://www.ata.org/understanding-facts).

The problem of chronic tinnitus is mainly of central origin. The pathophysiological substrate of chronic tinnitus is still a matter of intense debate (De Ridder et al., 2014b; Norena, 2015). The majority of chronic states of tinnitus begin with the functional damage of the cochlea (i.e., noise or hearing loss) and consequent abnormal input to the higher level neural structures of the auditory pathway (Eggermont and Roberts, 2004; Guitton, 2012; Chen et al., 2013; Wu et al., 2016). When tinnitus stabilizes as a chronic condition (not less than 1 year, Malinvaud et al., 2016), then numerous functional disarrangements occur, like the hyperpolarization of thalamic relay cells (Llinas et al., 2005), the changes in the central neural sensitivity or gain (Norena, 2011) and the functional coupling of different parallel brain networks (Kalauzi et al., 2012, 2014) into the “tinnitus (sub)network” (De Ridder et al., 2014b). This finally results also with the functional and anatomical disarrangement of the auditory cortex tonotopic map (Norena and Eggermont, 2005; Guitton, 2012).

The central (Zoccoli et al., 2005; Bojić et al., 2016) and peripheral components of the autonomic nervous system (Bojić, 2003; Silvani et al., 2003; Platiša et al., 2016) play crucial roles in the stabilization and manifestations of chronic tinnitus (Jastreboff, 2011). Cholinergic innervation plays a major role in the development and plasticity of the auditory cortex (Shideler and Yan, 2010). The growth and functional coupling of cholinergic innervations with auditory cortical cells goes in parallel with the forming of thalamocortical connections during embryological development and in the early stage after the birth. The presence of muscarinic acetylcholine receptors plays a crucial role in this process: muscarinic receptor type 1 (M1) regulates the expression of different neurotrophins (brain-derived neurotrophic factor and nerve growth factor; Da Penha Berzaghi et al., 1993; Betancourt et al., 2006), determines the structure of neurons by promoting cell survival (Tobin and Budd, 2003) and stimulates the neural (VanDeMark et al., 2009) and dendritic outgrowth (Zhang et al., 2005). Finally, muscarinic antagonists decrease the frequency specific plasticity of the auditory cortex in the paradigm of auditory fear conditioning (Ji et al., 2005; Ji and Suga, 2009) and, importantly for this concept, the stimulation of nc. basalis Meynerti (Bakin and Weinberger, 1996; Weinberger, 2003). Nc. basalis Meynerti electrical stimulation paired with tones acutely enhances cortical neural plasticity and reverses the neurological and perceptual correlates of tinnitus in adult animals (Nichols et al., 2011). M1 impacts also the processes of learning and memory (Zhang et al., 2006; Butcher et al., 2016) which are crucially important for the genesis and maintenance of tinnitus (De Ridder et al., 2014b; Eggermont and Kral, 2016; Vanneste and De Ridder, 2016).

Different techniques, both noninvasive and invasive, were tested in order to reverse the pathological neuroplastic changes of the auditory cortex toward the physiological state (Vanneste and De Ridder, 2012). Vagus nerve stimulation paired with tone stimulation is among the most promising tools for treatment of chronic tinnitus (Engineer et al., 2011; Shetake et al., 2012; De Ridder et al., 2014a). It is based on the principle of provoking the central neuromodulatory responses by stimulation of vagal afferent fibers and nc. basalis Meynerti which abundantly innervate the auditory cortex. Vagus stimulation paired with tone stimulation has an effect of targeted plasticity, the phenomenon of reversing the map changes in individuals with tinnitus. The area of tinnitus specific cortical neurons is consequently diminished (Engineer et al., 2011). In human studies the efficiency of this method to reduce tinnitus severity was around 40%, and interestingly, all the patients who did not experience an improvement were on drug therapy that included, among others, muscarinic antagonists (De Ridder et al., 2014a). More, it is well-documented in the literature that M1 receptors have a crucial role in the experience-dependent plasticity of the auditory cortex (Shideler and Yan, 2010). Our hypothesis is that *in silico* identification of M1 receptor allosteric agonists would propose a new line for the clinical research for modulated vagus nerve stimulation (VNS) paired with tones, potentially more targeted and more efficient. In addition, by agonizing the cholinergic mechanisms of learning and memory, attention, stress response, wakefulness and sleep and sensory information processing (Ferreira-Vieira et al., 2016), M1 allosteric agonists would stabilize the process of re-learning of neural networks involved in tinnitus perception. In order to choose the best candidates for these drugs we applied *in silico* strategies that resulted with three candidates for M1 allosteric modulators.

## Methods

In this paper, we implemented a virtual screening protocol that includes both short and long-range interactions between interacting molecules. The long-range interactions are denoted by the parameters—the average quasi valence number (AQVN) and the electron-ion interaction potential (EIIP).

First, the EIIP/AQVN filter was applied for *in silico* screening of the DrugBank (http://www.drugbank.ca) (Wishart et al., 2006) and then followed by 3D QSAR and molecular docking for identification of candidate allosteric modulators of M1.

### EIIP/AQVN

The EIIP for organic molecules can be determined by the following simple equation derived from the “general model pseudopotential” (Veljkovic et al., 2011)

(1) $$\begin{array}{r} \text{EIIP}=0.25\;{\text{Z}}^{*}\text{ sin}\left(1.04\;\pi \;{\text{Z}}^{*}\right)/2\pi \end{array}$$

where Z^*^ is the average quasi valence number (AQVN) determined by

(2) $$\begin{array}{r} {\text{Z}}^{*}=\sum^{\text{m}}\left(\text{niZi}/\text{N}\right) \end{array}$$

where *Zi* is the valence number of the *i*th atomic component, *ni* is the number of atoms of the *i*th component, *m* is the number of atomic components in the molecule, and *N* is the total number of atoms. EIIP values calculated according to Equations (1, 2) are expressed in Rydberg units (Ry).

Further filtering of these compounds was performed by means of 3D QSAR(quantitative structure–activity relationships) in Pentacle software and followed by docking.

### 3D QSAR

In order to build a pharmacophoric model and select compounds based on their pharmacophoric similarity, 12 literature M1 modulators (Target ID CHEMBL216) were downloaded from the ChEMBL database. All compounds were converted into the SDF format and then imported, along with candidate compounds into the Pentacle QSAR software, protonated at pH 7.4, and oriented according to the principal moments of inertia. Standard GRIND descriptors were calculated and the PCA model was built. From PCA scores that included the first two major components, PC1 and PC2, the most similar drug molecules to literature allosteric modulators were selected for further filtering.

### Molecular docking

Molecular docking of selected candidates was performed into the built homology model of the M1 receptor. The binding site of allosteric modulators was identified from the 4MQT structure and was placed in between extracellular loops of the 2 and 3 region. The grid box for docking, with dimensions 20 × 20 × 20 Å was placed to occupy this space. The receptor and ligands were prepared in Autodock Tools 1.5.6. Docking was carried out in AutodockVina (Trott and Olson, 2010). Exhaustiveness was set to 250.

## Results

### EIIP/AQVN filter

**Figure 1:**
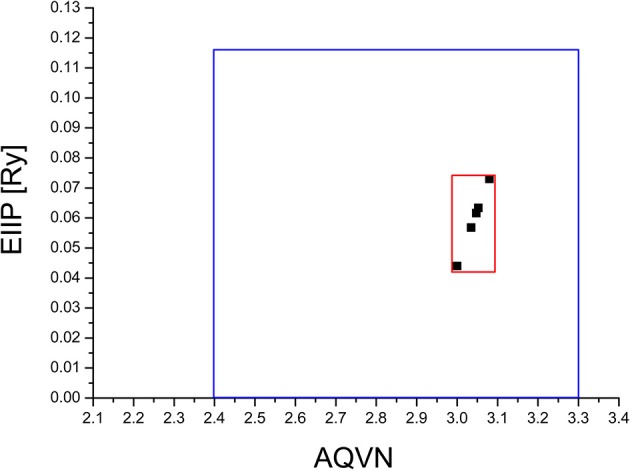
Schematic presentation of the EIIP/AQVN criterion for selection of candidate allosteric modulators of muscarinic receptor M1. Active domain (red): AQVN (3–3.08), EIIP (0.004–0.073). Chemical space (blue) AQVN (2.40–3.30) EIIP (0.000–0.116) EIIP/AQVN domain of homologous distribution of >90% compounds from PubChem Compound Database.

The virtual screening (VS) protocol in this study was based on the application of consecutive filters to select candidate allosteric modulators of M1. Previously it was shown for molecular targets in diverse pathological states that small molecules with similar AQVN and EIIP values interact with the common therapeutic agent (Veljkovic et al., 2011, 2013). This resulted in determining criteria for virtual screening of molecular libraries for compounds with similar therapeutic properties (Veljkovic et al., 2013). The selected learning set consisted of allosteric agonists and positive allosteric modulators of M1, reported in literature (Conn et al., 2009). The compounds from the learning set were inside the active domain with AQVN and EIIP values within the intervals of (3–3.08) and (0.004–0.073), respectively, and this domain was selected as a criterion for the selection of compounds representing candidate allosteric modulators of M1 (Figure 1). By applying the EIIP/AQVN-based virtual screening criterion, 50 drugs were chosen out of 1463 approved drugs from the DrugBank (http://www.drugbank.ca) (Wishart et al., 2006).

### QSAR selection

The 103 molecules after EIIP filtering were subjected to pharmacophoring modeling and selection. Twelve literature M1 modulators were converted into 3D structures, along with drug candidates and GRIND descriptors for all compounds were calculated, from which a PCA model was built (Table 1).

**Table 1:** PCA models for M1 alosteric modulators and candidates.

**Component**	**SSX**	**SSXacc**	**VarX**	**VarXacc**
1	44.48	44.48	43.92	43.92
2	11.32	55.8	10.98	54.9
3	5.48	61.29	5.19	60.09
4	4.97	66.26	4.77	64.86
5	4.09	70.35	3.94	68.8

*SSX, Percentage of the X sum of squares; SSXacc, accumulative percentage of the X sum of squares; VarX, percentage of the X variance; VarXaac, accumulative percentage of the X variance*.

From PCA scores that included the first two major components, PC1 and PC2, based on their vicinity in the PC1-PC2 space to literature M1 modulators, the most pharmacologically similar drug molecules were selected. The QSAR filtering was carried in Pentacle software (Duran et al., 2009). Finally, 10 compounds were selected for molecular docking (Table 2).

**Table 2:** QSAR selection of 10 compounds for molecular docking, with docking energies.

**Compound**	**VINA docking energy, kcal mol-1**
Estazolam	−9.9
Diloxanide	−9
Flumazenil	−9.2
Rosoxacin	−9.6
Bromazepam	−9.3
Penicilin V	−9
Sulfamethazine	−8.4
Fluconasole	−8.7
Dapsone	−8
Bromfenac	−9.4
Raltegravir	−10.3

### M1 muscarinic receptor homology modeling

In order to obtain the relevant structure of the M1 receptor active state, the crystal structure of the M2 muscarinic receptor in the active state with the agonist and allosteric modulator (PDB ID 4MQT) was used as a template for homology modeling. The P11229 sequence of the Homo sapiens muscarinic acetylcholine receptor M1 was used. The modeling was carried out on the Protein Homology/analogY Recognition Engine V 2.0 (Phyre) server (Kelley et al., 2015).

### Molecular docking

**Figure 2:**
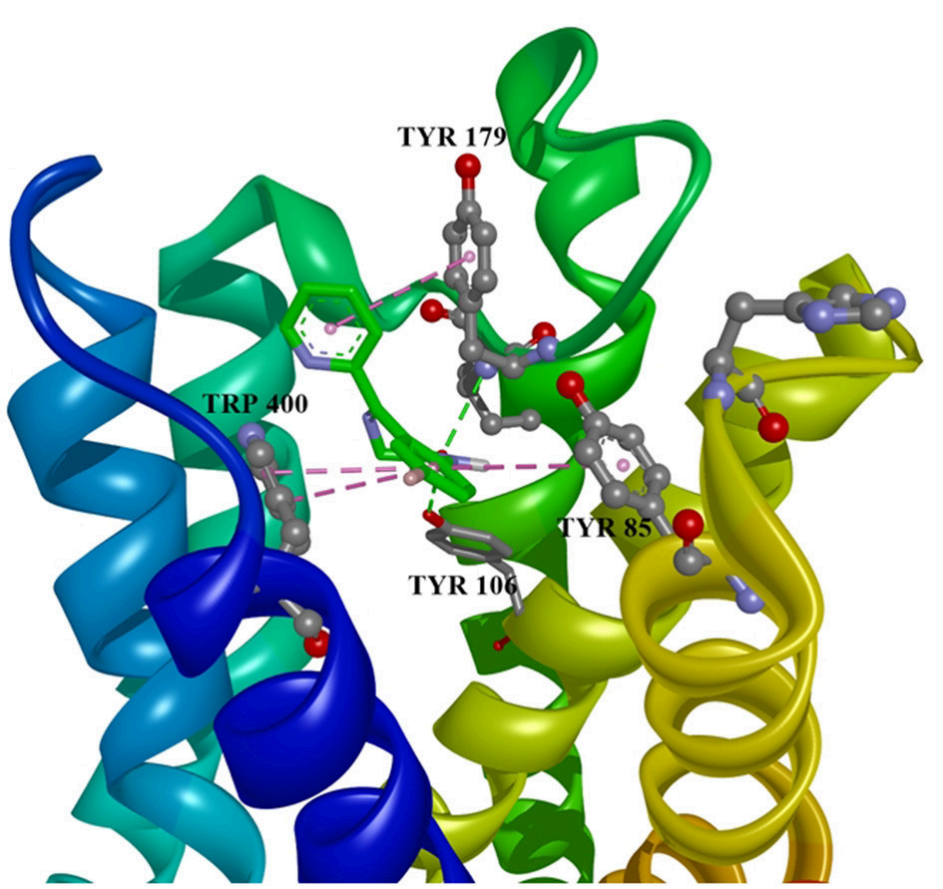
Bromazepam in allosteric modulator binding region of M1 acetylcholine receptor, with marked aminoacids, and intermolecular interactions. Green: hydrogen bonds, purple: hydrophobic interactions.

**Figure 3:**
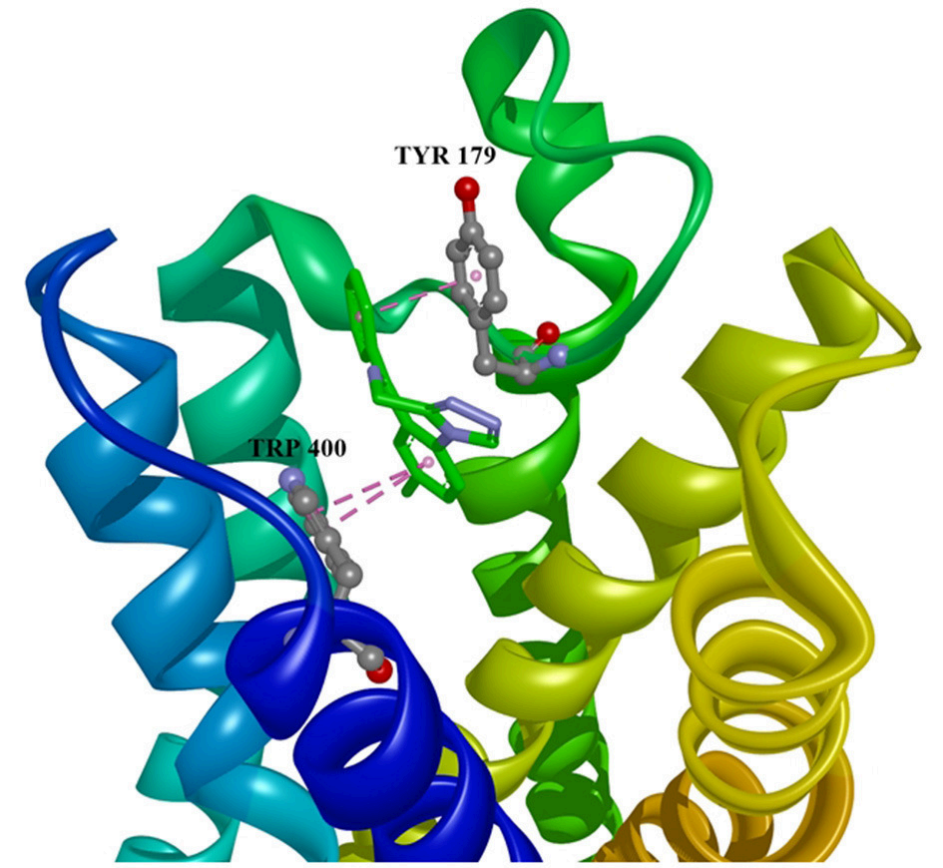
Estazolam in allosteric modulator binding region of M1 acetylcholine receptor, with marked aminoacids, and intermolecular interactions. Green: hydrogen bonds, purple: hydrophobic interactions.

**Figure 4:**
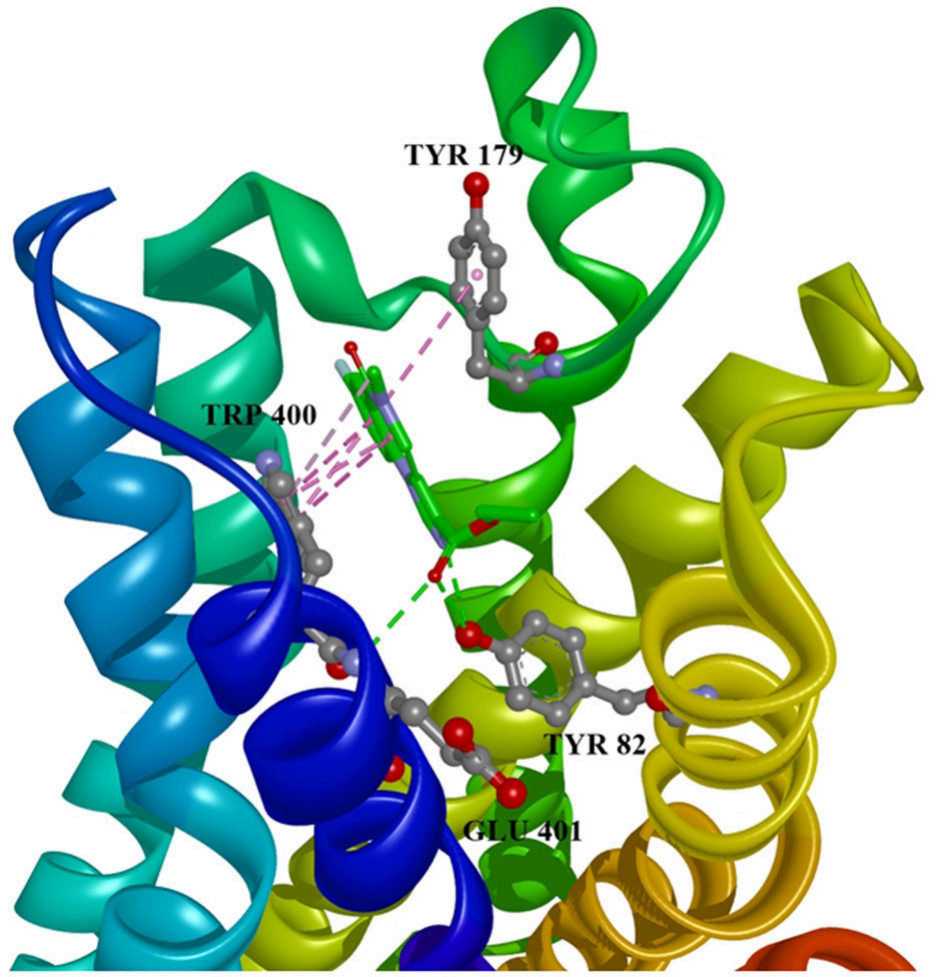
Flumazenil in allosteric modulator binding region of M1 acetylcholine receptor, with marked aminoacids, and intermolecular interactions. Green: hydrogen bonds, purple: hydrophobic interactions.

Ten selected compounds after 3D QSAR were further subjected to molecular docking into the M1 receptor model. Three compounds with the lowest binding energy values (Table 2) and identified hydrophilic and hydrophobic interactions with allosteric binding site amino acid residues (Tyr 82, Tyr 85, Tyr 106, Tyr 179, Trp 400, Glu 401) were selected to be the best candidates, along with considerations of drug side effects and purpose. Finally, BROMAZEPAM, ESTAZOLAM, and FLUMAZENIL were found to be the best promising candidates (Figures 2–4, respectively).

## Discussion

We hypothesized that allosteric activation of M1 receptors in the circumstances of increased cholinergic neurotransmission to the auditory cortex by targeted VNS, will provoke an increase of M1 mediated effects:

1. Neuromodulatory action of BDGF and NGF (Da Penha Berzaghi et al., 1993) in neuronal zones adjacent to the tinnitus;
2. Neuromodulatory action on frequency specific neuronal zones enabling the recruitment of pathologically active neurons back to the physiological pattern of activity;
3. Regaining the physiological tonotopy (Zhang et al., 2006) of the auditory cortex;
4. Recruitment of novel neurons from undifferentiated neuronal pools in the hippocampus (VanDeMark et al., 2009) and
5. The dendritic and axonal “rewiring” of the tinnitus “subnetwork” (De Ridder et al., 2014b).

The AQVN/EIIP approach was applied previously for the selection of new candidates in pharmacotherapy of the vasovagal syncope (VVS) revealing that the majority of antimuscarinic drugs might have a therapeutical potential for VVS. In this study sequential virtual screening criteria were applied, first the AQVN/EIIP based filter for the selection of candidate allosteric agonists of the M1 receptor selecting 50 drugs out of 1463 approved drugs from the DrugBank (http://www.drugbank.ca; Wishart et al., 2006). This step was followed by 3DQSAR analysis and finally docking. The screening identified bromazepam, estazolam, and flumazenil as the most promising drugs which could be repurposed as allosteric agonists of the M1 receptor.

Bromazepam and estazolam are known anti-anxiety agents with a hypnotic effect. Flumazenil has specific antibenzodiazepine action and is used as an antihypnotic agent in circumstances where the benzodiazepine and nonbenzodizepine hypnotic effect has to be reduced.

If we expand the concept to other methods of treatment of tinnitus (invasive or noninvasive; Vanneste and De Ridder, 2012), it is reasonable to propose that pharmacological facilitation of neuromodulatory changes by the *in silico* identified drugs in our investigation in tinnitus network structures would be beneficial even in these treatments. Future clinical studies of the combined M1 allosteric agonist therapy with VNS and potentially other invasive and noninvasive methods of chronic tinnitus treatment will reveal the final answer regarding their synergistic action.

Learning during wakefulness induces neural plasticity changes. Their stabilization occurs during sleep (Tononi and Cirelli, 2005). The process of sleep selects the synaptic weights of the neural synapses on the basis of their engagement during wakefulness: if the synapses are more used, the sleep process will make them more stabile while the less used synapses during wakefulness will not be strengthened. This is at least partially due to the intensity of cholinergic neurotransmission that increases from wakefulness toward NREM and REM sleep (Hobson and Friston, 2012). The last phase, REM, also known as a dream state, is the state of consciousness where tinnitus is not perceived (De Ridder et al., 2014b). The sleep process in this way supports the mechanisms of learning and memory acquired during wakefulness. If we presume that VNS paired with tones is also the process of re-learning of the cortical networks pathologically changed in tinnitus, then the quality of sleep in chronic tinnitus patients is essential for stabilization of daily acquired neuroplastic changes by the VNS therapy. In line with that, the cholinergic effect together with the hypnotic effect of the proposed drugs could be beneficial and their application justified before bedtime. The caveat that has to be taken into consideration is the feature of benzodiazepines to promote or to reinforce already existing sleep apnea by increased miorelaxation. This effect could diminish the amount of both NREM and REM sleep and potentially prevent the beneficial effect of the sleep on the process of the recovery in tinnitus patients. Biological experiments will address the question of concentration applied to the treatment of tinnitus and the question if sleep apnea as the contraindication for application of the proposed M1 allosteric agonists.

In conclusion, the results presented here suggest a potential novel approach in treatment of chronic tinnitus by VNS paired with tones with *in silico* repurposed drugs as therapeutic candidates that may target M1 and prospectively improve the treatment.

## Author contributions

TB, VP, MS, and SG contributed equally to the conception of the work, acquisition, analysis, and interpretation of data. TB, VP, and SG participated in drafting the manuscript, revisiting it critically and gave final approval of the version to be published. The authors reached the agreement to be accountable for all the aspects of the work in ensuring that questions related to the accuracy or integrity of any part of the work are appropriately investigated and resolved.

### Conflict of interest statement

The authors declare that the research was conducted in the absence of any commercial or financial relationships that could be construed as a potential conflict of interest.
